# Large-Scale Functional Brain Network Abnormalities in Alzheimer’s Disease: Insights from Functional Neuroimaging

**DOI:** 10.3233/BEN-2009-0227

**Published:** 2009-10-21

**Authors:** Bradford C. Dickerson, Reisa A. Sperling

**Affiliations:** ^1^Department of NeurologyHarvard Medical SchoolBostonMAUSA; ^2^Massachusetts Alzheimer’s Disease Research CenterHarvard Medical SchoolBostonMAUSA; ^3^Frontotemporal Dementia UnitHarvard Medical SchoolBostonMAUSA; ^4^Athinoula A. Martinos Center for Biomedical ImagingMassachusetts General Hospital and Harvard Medical SchoolBostonMAUSA; ^5^Division of Cognitive and Behavioral NeurologyDepartment of NeurologyBrigham & Women’s HospitalBostonMAUSA

**Keywords:** Alzheimer’s disease, mild cognitive impairment, functional magnetic resonance imaging, hippocampus, parietal cortex

## Abstract

Functional MRI (fMRI) studies of mild cognitive impairment (MCI) and Alzheimer’s disease (AD) have begun to reveal abnormalities in large-scale memory and cognitive brain networks. Since the medial temporal lobe (MTL) memory system is a site of very early pathology in AD, a number of studies have focused on this region of the brain. Yet it is clear that other regions of the large-scale episodic memory network are affected early in the disease as well, and fMRI has begun to illuminate functional abnormalities in frontal, temporal, and parietal cortices as well in MCI and AD. Besides predictable hypoactivation of brain regions as they accrue pathology and undergo atrophy, there are also areas of hyperactivation in brain memory and cognitive circuits, possibly representing attempted compensatory activity. Recent fMRI data in MCI and AD are beginning to reveal relationships between abnormalities of functional activity in the MTL memory system and in functionally connected brain regions, such as the precuneus. Additional work with “resting state” fMRI data is illuminating functional-anatomic brain circuits and their disruption by disease. As this work continues to mature, it will likely contribute to our understanding of fundamental memory processes in the human brain and how these are perturbed in memory disorders. We hope these insights will translate into the incorporation of measures of task-related brain function into diagnostic assessment or therapeutic monitoring, which will hopefully one day be useful for demonstrating beneficial effects of treatments being tested in clinical trials.

## 1. Introduction

Alzheimer's disease (AD) is the most common cause of dementia [60]. Typically, the symptoms of the disease begin with insidious episodic memory difficulties after the sixth decade of life and progress gradually toward prominent impairment in memory, executive function, visuospatial abilities, language, and other domains of cognition and behavior. Early in the course of the illness and often persisting throughout the illness, social skills and comportment are relatively preserved. Eventually, cognitive impairments interfere with complex activities of daily life and ultimately result in the loss of independent function. Treatments are symptomatic, in that clinical trials demonstrate short-term benefits in cognitive function but not a slowing of the rate of decline [20]. Increasing emphasis is being placed on the development of disease-modifying

ISSN 0953-4180/09/$17.00 © 2009 – IOS Press and the authors. All rights reserved therapies to impede the underlying neurodegenerative process of AD and thereby slow the rate of cognitive decline, hopefully preserving independent function.

By the time AD dementia is typically diagnosed, substantial neuronal loss and neuropathologic change have damaged numerous brain regions. Although it may be possible to reverse some aspects of this damage, it would be ideal to initiate treatment with neuroprotective medications at a time when – or even before – AD is mildly symptomatic, ideally prior to dementia [25]. To approach this goal, our capability needs to be improved to identify individuals with very mild symptoms of the disease while they are still largely independent in daily function [28]. Currently, individuals are often classified as having mild cognitive impairment (MCI) when symptoms suggestive of AD are present but mild enough that traditional diagnostic criteria (which require functional impairment consistent with dementia) are not fulfilled. This gradual transitional state may last for a number of years [3,31], and diagnostic criteria have been developed and operationalized [45,77]. Efforts are currently underway by international groups of experts to revise the diagnostic criteria for AD with the goal of diagnosis prior to dementia – one proposed criteria set already makes explicit use of imaging and cerebrospinal fluid biomarkers [36].

Although the medial temporal lobe (MTL) is thought to be the site of early pathology underlying the initial amnesic syndrome, pathologic alterations are detectable in other limbic and heteromodal cortical regions in AD [4,10,105], including very mild [14, 30] or prodromal [5,16,97,111] phases. The nature of symptoms and signs of the illness, as well as the selective involvement of multiple cortical “convergence zones” [47] have led to the conceptualization of AD as a disease of multiple large-scale brain networks. Episodic memory, executive functions, semantic and lexical retrieval, and visuospatial construction and praxis depend on the integrity of large-scale cortical and subcortical networks [69,70], each with nodes or hubs centered in limbic archicortical, allocortical and periallocortical cortex and heteromodal isocortex [13]. The predilection of AD for certain of these hubs produces the concurrent and parallel loss of abilities subserved by these networks.

Since the loss of mnemonic and cognitive abilities is an early clinical feature of AD, it is critical to further our understanding of abnormalities of the function of the large scale networks subserving these abilities early in the course of AD. One promising technique for this purpose is functional magnetic resonance imaging (fMRI), which is thought to provide an *in vivo* correlate of neural activity, particularly local field potential afferent processing in a given region of task-induced “activation” [63]. Newer analytic approaches to fMRI data are beginning to raise questions about areas of functional “deactivation” (in which task is associated with decreases in activity below the baseline level) and areas with cross-correlated functional activity, thought to represent functional networks [41,84]. Given the growing body of evidence that alterations in synaptic function are present very early in the disease process, possibly long before the development of clinical symptoms and even significant neuropathology [20,91], fMRI may be particularly useful for detecting alterations in brain function that may be present very early in the course of AD. In this article, we will review fMRI data regarding functional abnormalities in MCI and AD, with an emphasis on large-scale networks subserving memory.

## 2. Functional MRI: Strengths and weaknesses

Since functional neuroimaging tools assess inherently dynamic processes that may change over short time intervals in relation to a host of factors, these measures have unique characteristics that may offer both strengths and weaknesses as potential biomarkers of neurologic disease. Functional neuroimaging measures may be affected by transient brain and body states at the time of imaging, such as arousal, attention, sleep deprivation, sensory processing of irrelevant stimuli, or the effects of substances with pharmacologic central nervous system activity. Imaging measures of brain function may also be more sensitive than structural measures to constitutional or chronic differences between individuals, such as genetics, intelligence or educational level, learning, mood, or medication use. While these may be effects of interest in certain experimental settings, they need to be controlled when the focus is on disease-related changes between groups of patients and controls or within individuals over time.

Among functional neuroimaging techniques, fMRI has many potential advantages in studying patients with neurodegenerative disorders, as it is a non-invasive imaging technique that does not require the injection of contrast agent. It can be repeated many times over the course of a longitudinal study and thus lends itself well as a measure in clinical drug trials. It has relatively high spatial and temporal resolution, and the use of event-related designs enables the hemodynamic corre-

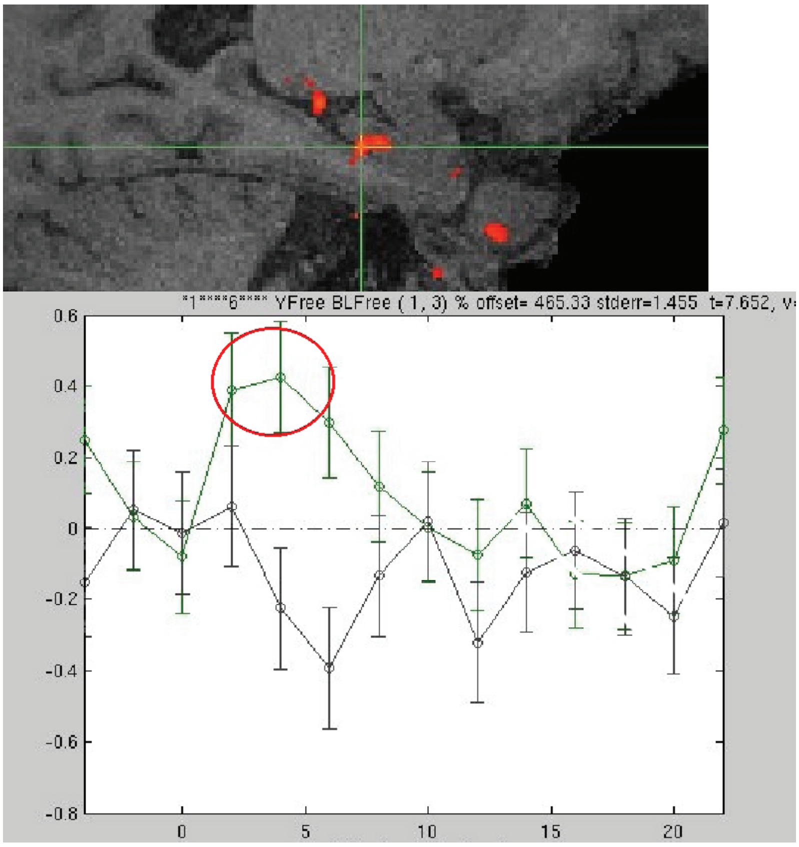

Fig. 1. High-resolution (2 mm$^3$) 3T fMRI data from a 76 year-old cognitively intact individual, showing hippocampal activation (top) during encoding that predicted successful subsequent free recall (circled peak activity in timecourse at bottom) as compared to items encoded but not recalled (uncircled timecourse).

lates of specific behavioral events, such as successful memory formation [32], to be measured.

There are, however, significant challenges to performing fMRI studies in cognitively impaired patients. The technique is particularly sensitive to even small amounts of head motion. Differences in task performance between patient and control groups complicate data interpretation [82]. Disease-related alterations in brain structure may make it difficult to interpret the source of abnormalities in functional data (i.e., simply a reflection of structural changes as opposed to primary functional changes). These issues pose non-trivial analytic challenges (e.g., structural-functional coregistration; multi-subject co-registration). Finally, it is critical to complete further reliability experiments if fMRI is to be used in longitudinal or pharmacologic studies. Although there are now a few studies of fMRI test-retest reliability in young subjects [65,67,100], reproducibility studies are only beginning to be performed in MCI and AD patients.

## 3. Large-scale brain networks supporting normal memory function

Functional neuroimaging has made valuable contributions to the cognitive neuroscientific investigation of brain networks subserving episodic memory processes in normal individuals. Multiple fMRI studies using a "subsequent memory" paradigm have demonstrated that greater fMRI activity during encoding in specific brain regions is associated with the likelihood of subsequent successful retrieval of the information [11,17, 58,99,109]. Regions within the MTL, prefrontal cortex (particularly the left inferior prefrontal cortex), and ventral temporal cortex have consistently demonstrated this subsequent memory effect (Fig. 1). Several pharmacological fMRI experiments have demonstrated decreased activation in the hippocampus and prefrontal regions with the administration of medications that impair memory performance, such as benzodiazepines and anticholinergics [89,103].

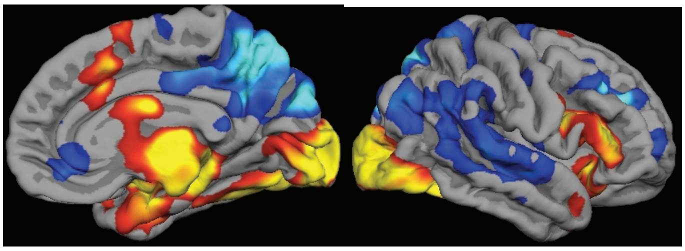

Fig. 2. Cortical regions in which activity is increased during successful encoding of new items (yellow/red) and in which activity is decreased during successful encoding of new items (blue). Increased and decreased activity is measured with respect to visual fixation. Regions in which task-induced deactivations are present represent the so-called default mode network.

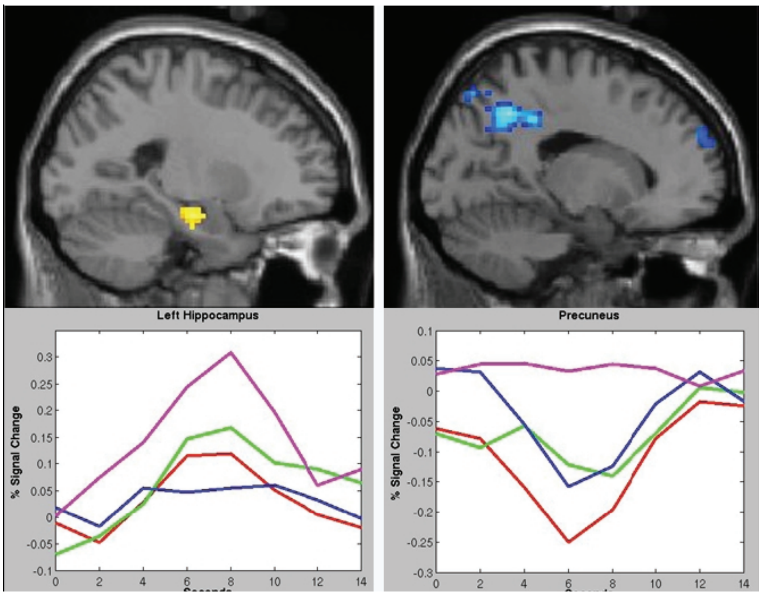

Fig. 3. During encoding, hippocampal activation (top left) accompanied by medial parietal deactivation (top right) are both important predictors of memory performance. Low performing elderly (maroon line in bottom graphs) individuals fail to deactivate the precuneus (bottom right) and demonstrate increased hippocampal (bottom left) and prefrontal activation for successful but not failed encoding trials, perhaps as a compensatory response to failure of default network activity [72]. Other colors on response curves are high-performing elderly (blue), low-performing young (green), and high-performing young (red).

In addition to regions of greater activation during memory performance, functional neuroimaging techniques have also illuminated a consistent set of brain regions that "deactivate" (i.e. demonstrate a decrease in BOLD activity with task) during successful memory formation [23,72]. These regions, in particular, lateral parietal and medial parietal regions, including the precuneus and posterior cingulate, are central components of the "default-mode network" (Fig. 2), characterized by Raichle and colleagues in a series of both PET and fMRI studies [41,83].

These parietal regions demonstrate functional connectivity with the MTL in resting-state network analyses [43,107]. Some of these parietal regions typically activates during memory retrieval tasks [14,108,110]. The degree to which individuals can deactivate this network during encoding appears to be strongly related to their subsequent memory performance [72,98] (Fig. 3). Thus, our current hypothesis is that successful memory formation requires coordinated and reciprocal activation in the hippocampal nodes of the episodic memory system and deactivation in the retrospenial-parietal nodes of this system.

## 4. FMRI in MCI and AD

Functional MRI has been used to investigate abnormalities in patterns of regional brain activation during a variety of cognitive tasks in patients diagnosed with

AD compared to control subjects. It is important to keep in mind that the abnormalities found in an fMRI study of an AD or other patient group are heavily dependent on the type of behavioral task used in the study – if the task does not engage a given brain circuit, functional abnormalities will not likely be observed in that circuit even if it is affected by the disease. Also, the nature of functional abnormalities may depend on whether the activated brain regions are directly affected by the disease, are indirectly affected via connectivity, or are not pathologically affected. Analytic and visualization software tools are now available to directly investigate the overlap of disease-related alterations in brain structure and task-related functional activity, but further efforts in computational and visualization software development are essential. It should also be kept in mind that even brain regions not usually thought to be affected by AD (sensorimotor areas) have been shown to exhibit abnormal function in AD patients [12, 21].

### *4.1. Abnormalities in activation of the medial temporal lobe memory system in AD and MCI*

With respect to memory, a number of fMRI studies in patients with clinically diagnosed AD, using a variety of visually presented stimuli, have identified a lesser degree of activation in hippocampal and parahippocampal regions compared to control subjects during episodic encoding tasks [56,66,87,94,101]. AD patients have also demonstrated increased activation in MTL regions to repeated or highly familiar stimuli, which may represent a failure of the normal repetition suppression response [42,81]. Neocortical abnormalities in AD have also been demonstrated using fMRI, including decreased activation in temporal and prefrontal regions. In addition to AD-related differences in task-related blood-oxygen level dependent (BOLD) signal amplitude or spatial extent, the temporal dynamics of activation appear to be altered in patients with AD [88]. And, as observed in other types of tasks, *increased* activation in prefrontal and other regions has also been found in AD patients performing memory tasks [101].

A recent quantitative meta-analysis [90] of both fMRI and FDG-PET memory activation studies of AD identified several regions as consistently being more likely to show greater encoding-related activation in controls than in AD patients, including hippocampal formation, ventrolateral prefrontal cortex, precuneus, cingulate gyrus, and lingual gyrus. Controls were more likely to show greater retrieval-related activation than AD patients in frontopolar, medial prefrontal, superior parietal, precuneus, superior temporal, amygdala, and parahippocampal regions. Compared to controls, AD patients showed greater likelihood of encoding-related activation in ventrolateral prefrontal, orbitofrontal, dorsolateral prefrontal, superior temporal, and fusiform regions. Greater retrieval-related activation was more likely in AD patients than in controls in dorsolateral prefrontal, ventrolateral prefrontal, precuneus, and supramarginal gyri.

Although AD patients consistently demonstrated a lesser degree of MTL activity than controls, they consistently (across multiple studies) demonstrated some degree of right parahippocampal activation during encoding, indicating that MTL brain regions are not entirely unable to generate memory-related activity. Furthermore, there was consistent hypoactivation in frontopolar activation in AD compared to controls during both encoding and retrieval, but consistent hyperactivation in dorsolateral and ventrolateral prefrontal regions, suggesting the presence of both dysfunction and possibly compensation in functional brain networks in AD. Finally, regions of the cognitive control network (dorsolateral prefrontal, posterolateral parietal, anterior cingulate, frontoinsula) were not engaged as robustly in AD as in controls, indicating the contribution of dysfunction in other cortical networks to impaired memory function in AD.

Several groups have also reported alterations in the pattern of deactivation in AD patients [14,44,64,79,81, 86]. These alterations in deactivation occur in regions of the so-called default mode network [83], which overlap substantially [14] with brain regions in which fibrillar amyloid deposition is detected with Pittsburgh Compound B (PIB) in PET studies in AD [59], as well as to the pattern of hypometabolism found on FDG PET studies of AD patients [1,68,92] and subjects at-risk for AD [51,85,93]; and of hypoperfusion on resting MR perfusion studies in AD [2,52]. In addition, the default mode network has demonstrated alterations at rest and in block-design fMRI paradigms in aging and AD [44, 64].

It appears that alterations in hippocampal activation and parietal deactivation over the course of MCI and AD are strongly correlated [15]. Similarly, resting state fMRI data has demonstrated alterations in parietal and hippocampal connectivity in MCI and AD [44]. Thus, converging evidence suggests that a distributed memory network is disrupted by the pathophysiological process of AD, which includes both medial temporal lobe systems and medial and lateral parietal regions involved in default mode activity. Future studies to probe alterations in connectivity between these system, which combine fMRI with other techniques such as diffusion tensor imaging, may prove particularly valuable in elucidating the early functional alterations in AD [112].

With respect to task-related activation in MCI, a handful of fMRI studies have been published to date and the results, thus far, have been variable, with some studies identifying a lesser degree of MTL activation in MCI compared to controls [55,66,94]. Petrella et al. [80] found no differences between MCI and controls in MTL activation during encoding, but observed hippocampal hypoactivation in MCI vs. controls during retrieval. Hippocampal hypoactivation in MCI was no longer seen when memory performance accuracy was included as a covariate in the analysis. Johnson et al. used a paradigm involving the repetitive presentation of faces to demonstrate that MCI patients do not show the same slope of decreasing hippocampal activation with face repetition that is seen in older controls, suggesting disruption of this "adaptive" response in the medial temporal lobe [53].

Several studies have reported greater MTL activation in MCI patients compared to controls. We used an associative face-name encoding paradigm to compare MTL activation in very mild MCI, AD, and controls [34]. Compared with controls, MCI subjects showed a greater extent of hippocampal activation and a trend toward greater entorhinal activation. Furthermore, there was minimal atrophy of the hippocampal formation or entorhinal cortex in this MCI group. The AD patients had smaller MTL volumes and a lesser degree of activation in these regions, and performed below controls on the post-scan memory test. Across all the subjects in the three groups, post-scan memory task performance correlated with extent of activation in both the entorhinal cortex and hippocampus.

Using a visual object encoding paradigm, Hamalainen et al. found that MCI subjects had greater activation (than controls) of caudal hippocampal formation, parahippocampal gyrus, and fusiform cortex [47]. Based on MMSE and neuropsychological data, the MCI subjects in this study were on the relatively more impaired end of the MCI spectrum (although CDR-SB was still mildly impaired), yet the group performed the fMRI memory paradigm relatively well – better than the AD group – although not as well as controls. In the first event-related subsequent memory study of MCI, Kircher et al. used an item-based task with words and found that MCI subjects activated rostral left hippocampal and surrounding cortical regions to a greater degree than controls [57]. MMSE scores from these MCI participants suggested that the group was at the more impaired end of the MCI spectrum, but neuropsychological data indicated milder impairment – in fact, delayed verbal recall scores were minimally impaired relative to controls, with scores for the MCI participants ranging as high as 14 items freely recalled after a 20 minute delay in this 15-item test. In addition, the MCI participants performed similarly to controls on the fMRI memory paradigm. In an event-related verbal memory retrieval task, Heun and colleagues also found evidence of increased activation in MCI subjects compared to normal older controls when specifically examining successful retrieval trials [50].

The variability in fMRI data from MCI subjects probably relates, at least in part, to the complex relationships between the severity of the subjects' clinical impairment and to their ability to perform the memory task employed as the fMRI paradigm. In addition, the particular fMRI memory paradigms, scanning techniques, and analytic approaches likely contribute to this variability. These issues are discussed in detail elsewhere [30].

Despite all the caveats, there is replicated evidence to support the hypothesis that there may be a phase of increased MTL activation in MCI. This increase, which also may be present in cognitively intact carriers of the APOE-e4 allele (for review, see [112]), may represent an attempted compensatory response to AD neuropathology, given that some MCI individuals with smaller hippocampal volume perform similarly on memory tasks to MCI individuals with larger hippocampal volume but have relatively greater MTL activation [33,47]. Additional studies employing event-related fMRI paradigms [32,57,99] will be very helpful in determining whether increased MTL activation in MCI patients is specifically associated with successful memory, as opposed to a general effect that is present regardless of success (possibly indicating increased effort). It is possible that MTL hyperactivation reflects cholinergic or other neurotransmitter upregulation in MCI patients [26]. Alternatively, increased regional brain activation may be a marker of the pathophysiologic process of AD itself, such as aberrant sprouting of cholinergic fibers [49] or inefficiency in synaptic transmission [104]. It is important, however, to acknowledge that multiple non-neural factors may confound the interpretation of changes in the hemodynamic response measured by BOLD fMRI, such as age- and disease-related changes in neurovascular coupling [12,21], AD-specific alterations in vascular physiology [75], and resting hypoperfusion and metabolism in MCI and AD [37], which may result in an amplified BOLD fMRI signal during activation [18,24,40]. Further research to determine the specificity of hyperactivation with respect to particular brain regions and behavioral conditions will be valuable to better characterize this phenomenon.

### 4.2. *MTL hyperactivation as a predictive biomarker in MCI*

We recently extended a preliminary analysis of fMRI as a predictor of dementia in MCI [33]. Over a follow-up interval of more than 5 years after fMRI scanning in 25 MCI subjects some showed no change and others progressed to dementia (change in CDR-Sum-of-Boxes ranged from 0 to 4.5). The degree of cognitive decline was predicted by hippocampal activation at the time of baseline scanning, with greater hippocampal activation predicting greater decline [71]. This finding was present even after controlling for baseline degree of impairment (CDR-SB), age, education, and hippocampal volume. These data suggest that fMRI may provide a physiologic imaging biomarker useful for identifying the subgroup of MCI individuals at highest risk of cognitive decline for potential inclusion in disease-modifying clinical trials.

If, in fact, the “inverse U-shaped curve” of hyperactivation that we hypothesize takes place early in the course of prodromal AD (at the clinical stage of MCI) is confirmed by future longitudinal studies, then the use of fMRI as a physiologic imaging biomarker will have to grapple with the problem of “pseudonormalization” of activation when individuals with MCI demonstrate progressive decline that results in the loss of hyperactivation. It may be possible to use a combination of clinical (e.g., CDR Sum-of-Boxes), neuropsychologic (e.g., memory tasks), anatomic (e.g., hippocampal and/or entorhinal volume), and molecular (e.g., FDG-PET) measures to assist in the determination of where an individual is along the inverse U-shaped curve of MTL activation. That is, moderate hyperactivation in the setting of minimal clinical and memory impairment and relatively little MTL atrophy would be consistent with the upgoing phase of the hyperactivation curve while the same level of hyperactivation in the setting of more prominent clinical and memory impairment and MTL atrophy would be consistent with the downgoing phase of the curve. In the end, it will be critical to perform longitudinal studies to determine whether this model of the physiologic, anatomic, and behavioral progression of MCI is supported by trajectories in individuals and groups of subjects.

We have recently completed longitudinal fMRI studies in a group of 51 older individuals, across a range of cognitive impairment, imaged with alternate forms of the face-name paradigm at baseline and two-year follow-up [102]. Preliminary analyses indicate that subjects who remained cognitively normal over the 2 years demonstrated no evidence of change in activation, whereas the subjects who demonstrated significant cognitive decline demonstrated a decrease in activation, specifically in the right hippocampal formation. Interestingly, we again observed that those subjects who declined had greater hippocampal activation at baseline, and that the amount of hyperactivation at baseline correlated with both loss of hippocampal signal and amount of clinical decline over two years. Thus, although we have hypothesized that hippocampal hyperactivation may be compensatory, it may also be a harbinger of impending hippocampal failure.

## 5. fMRI studies in asymptomatic subjects at elevated genetic risk for AD

Asymptomatic individuals with genetic risk factors for AD, such as carriers of the apolipoprotein E episilon 4 allele or autosomal dominant mutations such as presenilin 1, are particularly important subjects to assess with functional imaging. Bookheimer and colleagues [8] reported that, despite equivalent performance on a verbal paired-associate task, cognitively intact ApoE e4 carriers showed significantly greater activation, particularly prominent in bilateral MTL regions, compared to non-carriers. Subsequent studies stratified by ApoE genotype have been somewhat mixed in their results, with several studies also reporting greater activation in ApoE e4 carriers [7,39,48,96,113] but there have also been a similar number of studies finding evidence of decreased activation in ApoE e4 carriers [9,61,62,74, 95,106].

A very recent resting state and memory task-related fMRI study of young individuals demonstrated that medial temporal, medial prefrontal, and medial parietal regions showed stronger cross-correlated activity in resting-state functional connectivity of the default mode network in APOE e4 carriers than in non-carriers. Furthermore, memory task activation was more prominent in ventromedial temporal and hippocampal regions in e4 carriers than non-carriers, despite equivalent memory performance [38].

Individuals with other genetic risk factors for AD have also been studied with functional neuroimaging. Haier et al. [46] reported FDG-PET evidence of *increased* MTL activation (hypermetabolism) during cognitive tasks in non-demented Down's syndrome patients. A recent fMRI study by Mondadori et al. also found evidence of increased activation, which was specific to the episodic memory paradigm, in a young asymptomatic carrier of the presenilin 1 mutation [73]. A middle-aged presenilin 1 mutation carrier who fulfilled criteria for amnesic MCI showed decreased task-related activation. This study parallels the findings across the continuum of impairment in MCI subjects discussed above, and again suggests that there may be a non-linear trajectory of fMRI activation that evolves over the course of prodromal AD [98].

In an exciting area of recent research, there have been several studies of individuals with a family history of AD but without known genetic abnormalities. Again, somewhat discrepant results have been reported. Compared to a control group without a family history of AD, adult children of autopsy-confirmed AD patients exhibited increased activation in the frontal and temporal lobes, including the hippocampus [6]. Although a large percentage of the at-risk subjects possessed at least one copy of the ApoE e4 allele, the increased activation was found to be unrelated to this genetic risk. Johnson and colleagues have conducted two large fMRI studies comparing asymptomatic middle-aged adults (mean age 55) who have a parent clinically diagnosed with sporadic AD versus matched controls without parental history of AD [54]. Both of these studies, one an encoding task and one a metamemory task, demonstrated *decreased* hippocampal activation in the higher risk group. Interestingly, although there was no main effect of ApoE genotype seen in these studies, the group with a negative family history but who did possess an ApoE epsilon 4 allele showed the *greatest* hippocampal activation. This group also performed at the highest level of accuracy in the recognition task. These studies, which stratify subjects by family history, suggest that there may be a complicated interaction between ApoE and other genetic risk factors that influence hippocampal activation. Furthermore, there is very recent evidence that both hippocampal and medial parietal activation during a memory task are altered not only by the presence of ApoE genotype but also by the presence of first-degree family history of AD, suggesting an interaction between these factors [114].

Again, the discrepant results in this area will likely benefit from longitudinal fMRI testing, ideally in combination with amyloid and FDG-PET imaging and detailed anatomic measurements. These types of studies will probably provide critical information to improve our understanding of the temporal sequence of events early in the course of AD.

## 6. Conclusions

FMRI is a particularly attractive method for studying cognitive task-related patterns of brain activation in MCI and AD. Despite the relative infancy of the field, there have already been a number of promising fMRI studies in AD, MCI, and related disorders which highlight the potential uses of fMRI in both basic and clinical spheres of investigation. FMRI may provide novel insights into the neural correlates of memory and other cognitive abilities, and how they are altered in AD and MCI. It may illuminate large-scale functional network abnormalities early in the course of the disease, including those that can be identified from relatively short "resting-state" acquisitions, which are much less cumbersome than task-related fMRI. Finally, fMRI measures hold promise for multiple clinical applications, including early detection and differential diagnosis, predicting future change in clinical status or cognitive performance, and as a marker of alterations in brain physiology related to potential therapeutic agents [27, 29]. The greatest potential of fMRI likely lies in the study of very early AD, at the point of subtle neuronal dysfunction. However, a number of challenges remain.

Since a definitive diagnosis of AD and related neurodegenerative diseases can only be made at autopsy, neuroimaging studies of these disorders face challenges related to clinicopathologic heterogeneity; this is particularly true for MCI. Although all patients with AD progress through some form of an MCI phase prior to dementia, the converse is not true. That is, some patients who fulfill MCI criteria may have non-AD disease states, such as mesial temporal sclerosis [78]. Furthermore, the rate at which individuals with MCI decline within this diagnostic category and ultimately develop dementia may vary considerably. Thus, although prodromal AD may be identifiable as MCI clinically [45], it is important to recognize the heterogeneity present within this clinical construct. Continued efforts to further refine clinical diagnostic [76] and staging methods [22,31] should help improve our understanding of the relationships between the characteristics of individuals with MCI and imaging data. Thus, while the data reviewed above indicates that fMRI is sensitive to clinical diagnosis, symptom severity, and memory performance abilities, the discrepancies highlight the need for further fMRI research in the context of rigorous clinical assessment, longitudinal follow-up, and ideally multimodal imaging (i.e., volumetric structural MRI, perfusion measures, and nuclear medicine studies of metabolism and pathology).

It is very important for the field of fMRI to continue to expand to include different types of tasks, such as challenging executive or language tasks, since very early semantic, conceptual, reasoning, and other deficits have been reported in MCI long before a diagnosis of AD dementia [3]. Further studies employing multimodal MRI data analysis – including structure-function analyses of both grey matter regions and white matter tracts as they relate to functional networks – will be valuable, especially given the spatially distributed but subtle atrophy that can be present early in the course of prodromal AD [5,30]. Finally, it will be critical to incorporate molecular imaging measures into the design and analysis of fMRI experiments. The technical hurdles involved in rigorous data analysis for multi-modal imaging data, such as co-registration and partial volume correction issues, are not trivial and teams working on these efforts deserve enthusiastic support from the community attempting to apply these tools.

In the end, we hope that the variety of maturing and emerging neuroimaging tools will contribute in fundamental ways to earlier diagnosis, prognosis and monitoring of progression, and measurement of putative treatment effects for this terrible disease that robs patients and families of their past and future.

## Acknowledgments

The authors would like to thank our patients, colleagues, and study staff for continued inspiration. Supported by grants from the NIA (R21-AG029840, R01-AG029411, R01-AG027435, P50-AG05134) and the Alzheimer's Association.
